# Prevalence of antiplatelet therapy in patients with diabetes

**DOI:** 10.1186/1475-2840-4-18

**Published:** 2005-12-01

**Authors:** Shaun R Miller, Benjamin Littenberg, Charles D MacLean

**Affiliations:** 1 University of Vermont College of Medicine, Burlington, Vermont, USA

## Abstract

**Objective:**

To determine the prevalence of, and patient characteristics associated with, antiplatelet therapy in a cohort of primary care patients with Type 1 or Type2 diabetes.

**Methods:**

Subjects participating in a randomized trial of a decision support system were interviewed at home and medication usage verified by a research assistant. Eligibility for antiplatelet therapy was determined by American Diabetes Association criteria and clinical contraindications. The association between antiplatelet use and patient characteristics was examined using bivariate and multivariate logistic regression.

**Results:**

The mean age of subjects was 64 years (range 31–93). The prevalence of antiplatelet use was 54% overall; 45% for subjects without known CVD vs. 78% for those with CVD; 46% for women vs. 63% for men; and 45% for younger subjects (age< 65) vs. 62% for senior citizens. After controlling for race/ethnicity, income, education, marital status, insurance status and prescription coverage, the following were associated with the use of antiplatelet therapy: presence of known CVD (OR 3.4 [2.2, 5.1]), male sex (OR 2.0 [1.4, 2.8]), and age > = 65 (OR 1.9 [1.3, 2.7]). The prevalence of antiplatelet therapy for younger women without CVD was 32.8% compared to a prevalence of 90.3% for older men with CVD.

**Conclusion:**

Despite clinical practice guidelines recommending antiplatelet therapy for patients with diabetes, there are still many eligible patients not receiving this beneficial therapy, particularly patients under 65, women, and patients without known CVD. Effective methods to increase antiplatelet use should be considered at the national, community, practice and provider level.

## Introduction

Cardiovascular disease (CVD) is the leading cause of morbidity and mortality in adults with diabetes [1-4]. Antiplatelet therapy, with either aspirin or the newer platelet aggregation inhibitors, has been shown to be safe and cost effective for reducing the risk of recurrent vascular events [5-8]. Consensus guidelines recommend the use of antiplatelet therapy for both primary and secondary prevention of CVD [9,10]. In 1997, the American Diabetes Association (ADA) recommended antiplatelet therapy for adults with diabetes and co-existing CVD, and for adults with diabetes over 30 years of age, even in the absence of CVD [11]. Prior to the publication of the ADA recommendations for antiplatelet prophylaxis, the national rate of aspirin use among patients with diabetes was estimated at 13% for individuals without CVD and at 37% for those with CVD [12]. By 2001 this latter prevalence, as determined by telephone survey, had increased to 48.7% [13]. Current estimates suggest that approximately 5% of adults cannot tolerate aspirin therapy. For these individuals, an alternative antiplatelet agent may be used [14].

Despite increasing evidence to support its effectiveness among patients with diabetes, antiplatelet therapy has been under-utilized [12,15,16], particularly in women [13]. While several observational studies have examined the prevalence of aspirin use both before and after the publication of the 1997 ADA recommendations, none have included the use of other antiplatelet agents and may therefore have underestimated the prevalence of antiplatelet therapy. The goal of this study is to determine the prevalence of antiplatelet therapy (aspirin and newer platelet aggregation inhibitors) for both primary and secondary prevention of CVD in diabetes and to examine the patient characteristics that are associated with failure to use this important therapy.

## Methods

This study was part of a larger project, the Vermont Diabetes Information System (VDIS), a cluster-randomized trial of a laboratory-based diabetes decision support system in a region-wide sample of 7295 adults with diabetes from 55 community Primary Care practices [17]. We did not distinguish between Type 1 and Type 2 diabetes because this distinction is not clinically important when recommending antiplatelet therapy. A field survey targeted at a sub-sample of subjects was designed to provide a better understanding of the non-laboratory features of diabetes. Patients were selected at random from the subjects in each practice participating in the VDIS trial and invited by phone to participate in an in-home interview. Patient names were randomly sorted and patients contacted by telephone until a sample of approximately 15% of the patients from each practice agreed to an interview. We attempted to contact 4209 patients and reached 1576. Of these, 1006 agreed to be interviewed. Demographic information including age, sex, race, ethnicity, education, income, marital status and history of cardiovascular disease were obtained by questionnaire. A complete list of medications was obtained by a research assistant by direct observation of all of the medication containers and recording of the medication name, dose, frequency and route of administration. The interviews occurred between July 2003 and March 2005. The University of Vermont Institutional Review Board approved the study and all subjects gave written informed consent to participate in the interview.

For the purposes of this cross-sectional study, a subset of interviewed subjects was created using inclusion and exclusion criteria based on the current American Diabetes Association (ADA) recommendations for the use of antiplatelet therapy [18]. The subset of subjects who were eligible for antiplatelet therapy consisted of all subjects in the VDIS interview cohort 30 years or older, and those under 30 years with a self-reported history of either coronary heart disease, stroke or transient ischemic attack, or peripheral vascular disease. For the purposes of the study we defined cardiovascular disease (CVD) as any of the above manifestations of vascular disease. We excluded patients with specific contraindications to antiplatelet therapy: peptic ulcer disease (144), severe liver disease (13), and those on current warfarin therapy (75), for whom decisions about concomitant use of antiplatelet therapy and anticoagulation would be individualized. No information was available about side effects or previous discontinuation of therapy was available. Some subjects had more than one exclusion; a total of 221 subjects were excluded for a final sample of 785 subjects. Antiplatelet use was defined as daily use of aspirin (at least 75 mg/day); clopidogrel; ticlodipine; or cilostazol; or a combination of aspirin and clopidogrel, ticlodipine, or cilostazol daily. The specific indication for the anti-platelet agent in each subject was not known.

We used logistic regression to test the bivariate association of anti-platelet use with variables that were potentially important based on previous research and clinical judgment, including age, sex, race/ethnicity, income, education, marital status, insurance status and pharmacy benefits, years since diagnosis of diabetes, smoking, body mass index, frequency of visits to primary care physician, specialist involvement in care (endocrinologist visit in the last year, attendance at a diabetes education class within the last year), and the various categories of CVD. Variables that demonstrated an association in bivariate modeling at a significance level of p < 0.1 were further examined with multivariate regression modeling in which insignificant (p > 0.05) variables were eliminated in a backward stepwise fashion.

## Results

The characteristics of the study population are presented in Table 1. The mean age was 64 years with half the population over age 65. Most graduated high school and fewer than 3% were uninsured. Most subjects were overweight or obese (89%), with 18% falling in the severely obese category (body mass index of 40 or greater). Twenty-six percent of the population had cardiovascular disease, with myocardial infarction being the most common manifestation in 16%.

**Table 1:** VDIS Subjects Eligible for Antiplatelet Therapy (N = 785)

**Characteristic**	**N**	**Mean or Prevalence**
Female	435	55%
Age, mean (SD) (range)	785	64 (11.8) (31–93)
Age > = 65	395	50%
Race/Ethnicity		
White, non-Hispanic	764	97.6%
Education		
High school graduate	599	77%
Marital Status		
Married or living as married	503	64%
Current smoker	125	16%
Endocrinology consult visit in past year	122	16%
Diabetes education visit in past year	173	23%
Insurance Status *		
None	21	2.7%
Private	472	60.5%
Medicare	447	57.6%
Medicaid	143	18.5%
Military	33	4.3%
Annual household income		
< $30,000	407	56.4%
Body Mass Index		
Normal (< 25)	85	10.9%
Overweight (25–29.9)	182	23.4%
Obese (> = 30)	510	65.6%
Very Obese (> = 40)	141	18.2%
Cardiovascular Disease §		
Any CVD	206	26.2%
Myocardial Infarction	127	16.2%
Stroke	68	8.7%
Peripheral Vascular Disease	61	7.8%
Number of prescription medications, mean (SD)	785	6.2 (3.5)
Number of MD visits in previous year, mean (SD)	785	1.5 (2.2)
Years since diagnosis of DM, mean (SD)	785	9.9 (10.1)
Antiplatelet therapy (aspirin or other)	421	53.6%
Aspirin only	371	47.3%
Non-aspirin platelet aggregation inhibitor only	20	2.5%
Aspirin and platelet aggregation inhibitor	30	3.8%
Antiplatelet therapy if known CVD	206	78.2%

* Subjects may have more that one type of insurance coverage

§Subjects may have more than one type of CVD

The prevalence of antiplatelet use was 53.6% (47.3% aspirin alone, 2.5% newer platelet aggregation inhibitor and 3.8% both) for all eligible subjects and 78.2% for subjects with known CVD. The characteristics associated with antiplatelet medication use are noted in Table 2. Male sex and older age are both associated with a two-fold increase in antiplatelet use (p < 0.001). Cardiovascular disease was associated with a three-fold increase in antiplatelet use, with MI showing a six-fold increase (p < 0.001). Other factors that were associated with anti-platelet agent use were: an endocrinology visit in the previous year (p = 0.004), and Medicare insurance coverage (p < 0.001).

**Table 2:** Bivariate Associations with Anti-Platelet Use in Adults with Diabetes

**Characteristic**	**Odds Ratio [95% CI]**	**P Value**
Male sex	2.0 [1.5, 2.7]	**<0.001**
Age > 65 years	2.0 [1.5, 2.6]	**<0.001**
Race/ethnicity, White, non-Hispanic	2.0 [0.8, 5.2]	0.15
Education: > HS graduate	0.8 [0.6, 1.1]	0.25
Married or living as married	1.0 [0.8, 1.4]	0.84
Smoker	0.8 [.05, 1.1]	0.16
Endocrinology consult within 1 year	1.8 [1.2, 2.7]	**0.004**
Diabetes education class within 1 year	1.4 [1.0, 2.0]	**0.06**
Insurance category		
None	0.28 [0.10, 0.78]	**0.02**
Private	1.15 [0.86, 1.53]	0.35
Medicare	1.88 [1.40, 2.51]	**<0.001**
Medicaid	0.98 [0.68, 1.4]	0.91
Military	1.78 [0.85, 3.70]	0.13
Income < $30,000	1.1 [0.8, 1.5]	0.55
Prescription coverage	0.9 [0.7, 1.1]	0.19
Body Mass Index		
BMI category (normal, overweight, obese)	0.8 [0.7, 1.0]	0.11
Obese (BMI > 30)	0.8 [0.6, 1.0]	**0.07**
Severe Obesity (BMI > 40)	0.6 [.04, 0.9]	**0.01**
Type of Cardiovascular Disease		
Any CVD	3.3 [2.4, 4.3]	**<0.001**
Myocardial Infarction	6.7 [4.0, 11.3]	**<0.001**
Stroke	2.1 [1.2, 3.5]	**0.008**
Peripheral Vascular Disease	3.1 [1.7, 5.8]	**<0.001**
Number of PCP visits in previous month	1.04 [0.97, 1.11]	0.26
Duration of diabetes (in years)	1.01 [1.00, 1.03]	0.13

In multivariable analysis, three characteristics remained independently associated with antiplatelet use while controlling for important covariates (see Table 3). Subjects with a history CVD were more likely to be on appropriate antiplatelet therapy (OR 3.4 [CI 2.2, 5.1]), as were subjects 65 or older (OR 1.9 [CI 1.3, 2.7]) and men (OR 2.0 [CI 1.4, 2.8]), (p < 0.001 for each). Table 4 indicates the prevalence of antiplatelet use in each of these patient subgroups. The lowest rates are among women under 65 without CVD (32.8%), and highest among older men with known CVD (90.3%).

**Table 3:** Multivariate Model of Characteristics Associated with Anti-Platelet Use in Adults with Diabetes*

**Characteristic**	**Odds Ratio [95% CI]**	**P Value**
CVD history	3.4 [2.2, 5.1]	<0.001
Senior (age > 65)	1.9 [1.3, 2.7]	<0.001
Male sex	2.0 [1.4, 2.8]	<0.001

*Controlling for race, income, education, marital status, insurance, prescription coverage

**Table 4:** Prevalence of Antiplatelet Use by Patient Characteristic

	Sex	Age	% on Antiplatelet therapy	N
CVD Absent	Female	Less than 65	33%	180
				
		65 or older	45%	151
			
	Male	Less than 65	49%	137
				
		65 or older	60%	111
		
CVD Present	Female	Less than 65	58%	33
				
		65 or older	77%	70
			
	Male	Less than 65	78%	41
				
		65 or older	90%	62

Among the 206 subjects with known CVD we found similar associations with antiplatelet use with age > 65 (OR 3.0 CI [1.4, 6.3]), and male sex (OR 3.3 [1.5, 7.1]).

## Discussion

We found a prevalence of antiplatelet therapy use among adults with diabetes of 53.6% (47.3% aspirin alone, 2.5% newer platelet aggregation inhibitor and 3.8% both), which is similar to the recent nationally representative telephone survey estimate of aspirin use of 48.7% by Persell [13]. Among patients with CVD we found a prevalence of antiplatelet therapy of 78.2%, compared to 74.2% by Persell [13].

We found the highest rates among subjects with CAD. Following the CAPRIE trial in 1996, which showed a slight advantage in secondary prevention of cardiovascular events for clopidogrel vs. aspirin, clopidogrel has been increasingly used both in addition to aspirin and as its replacement [19,20]. The newer platelet aggregation inhibitors are also increasingly used for acute coronary syndrome and after percutaneous coronary intervention [21]. The strong evidence for CAD indications is reflected in our findings that subjects with coronary artery disease were the most likely to be receiving antiplatelet therapy.

Our motivation in exploring the factors associated with antiplatelet agent use was to help identify subgroups that may be targeted for special efforts to increase antiplatelet therapy. We found that women, patients younger than 65, and those without CVD were less likely to be using antiplatelet therapy. On the other end of the spectrum, over 90% of men over 65 with CVD were taking antiplatelet therapy. This high level of use among those at the highest risk supports the achievability of the consensus guidelines. A recent meta-analysis including 287 studies and 135,000 patients at high vascular risk showed that antiplatelet therapy reduced serious vascular events (non-fatal MI, non-fatal stroke, vascular death) by 36 (SE 5) per 1000 patients treated for two years [22]. Assuming that we can move from our overall prevalence of antiplatelet therapy of 54% to our best rate of 90%, we estimate that another 13 serious vascular events per 1000 could be averted over two years. If this is projected to the 18.2 million adults with diabetes in the United States [23], we estimate that 238,000 serious vascular events could be averted.

Why are patients with diabetes not receiving antiplatelet therapy despite consensus guidelines? First of all, prescribers may feel there is some ambiguity regarding the role of aspirin in CVD primary prevention for patients with diabetes. For example, while the Primary Prevention Project, which randomized over 4000 diabetic and non-diabetic subjects with CVD risk factors to aspirin or no aspirin, was stopped early because of the beneficial effects of aspirin, in the subgroup with diabetes the benefits were smaller and not statistically significant. [24] This raises the question of potential differences in the role of antiplatelet therapy in diabetes. Secondly, even if prescribers agree with the guideline, there are other barriers to achieving perfect compliance. A qualitative study exploring reasons cited by physicians for not prescribing aspirin included: difficulties in applying generic guidelines to individuals, patient resistance to taking aspirin, prioritization of other issues in a time constrained visit, and communication problems in reviewing the medications of patients with stroke [25].

Why might women be less likely to be receiving antiplatelet therapy? Gender differences have been well documented in the diagnosis and treatment of heart disease [26] In addition, the effects of aspirin may be different in men and women; a recent study of primary prevention of CVD in almost 40,000 women over 45 years of age showed that, while stroke risk was lowered, myocardial infarction and overall cardiovascular mortality were not [27]. Physicians may be less enthusiastic about the evidence base supporting the use of antiplatelet therapy in women. For patients under age 65, physicians (and patients themselves) may not perceive the risks of CVD as high enough to warrant antiplatelet therapy.

We observed an association between the use of antiplatelet therapy and the type of CVD. Patients with a history of prior myocardial infarction were more likely to be on antiplatelet therapy (86%) than those with a history of peripheral vascular disease (77%) or cerebrovascular accident (CVA) (69%). Furthermore, only 54% of patients with CVA and no other CVD were using an antiplatelet agent. Antiplatelet therapy has been shown to reduce the risk of recurrent CVA by 11% to 15% in patients with prior ischemic stroke of non-cardiac origin and reduce the risk of stroke, MI, and vascular death, by 22% [28]. The extent to which stroke patients and their physicians avoid antiplatelet therapy due to risk of bleeding is not known. This lower use of antiplatelet therapy in stroke patients identifies an area for potential investigation and intervention to improve anti-platelet regimens in this patient population.

Health insurance coverage has been shown to be an important factor in the delivery of medical services. Increasing levels of health insurance have a positive correlation with the likelihood that an individual will receive appropriate preventive care [29]. In diabetes, poor insurance coverage has been associated with delayed or omitted preventive services [30]. We found that health insurance coverage was not an important predictor of anti-platelet therapy in patients of this cohort, but the level of health insurance coverage was high and subjects were under the care of a primary care provider suggesting good access to care. In the case of an expensive medicine like clopidogrel, lack of prescription drug coverage could contribute to lack of use. However, aspirin, which comprises the majority of the antiplatelet agents in our study, is a low cost, nonprescription medication.

This study has several limitations. Our population, while representative of patients receiving primary care in the rural Northeast may not be representative of all adults with diabetes in the U.S. We do not have information regarding allergies or side effects associated with antiplatelet medications. It is possible that eligible subjects were unable to tolerate therapy, though it is unlikely this would be the case in more than 5% of subjects. It is unlikely that medication intolerance would be correlated with age, sex or cardiovascular disease. We do not have information regarding the indication for aspirin use, though 98% of subjects reported low-dose aspirin use (< = 325 mg/d) suggesting prophylaxis. Our analysis does not indicate causality and the exact mechanisms promoting or deterring the use of recommended interventions is unknown.

There have been a variety of successful interventions directed at increasing the use of antiplatelet therapy for the prevention of CVD including: HMO-directed quality improvement efforts [31], intensive multifaceted case management [32], pharmacy-directed interventions [33,34], and electronic medical record reminder systems [35]. A VA study found that physician counseling was highly associated with antiplatelet therapy and suggested that this simple intervention could prevent many cardiovascular events and deaths [36]. There are many ways in which antiplatelet use can be increased; it is now a question of which approach can be most efficiently adapted in each clinical setting.

## Conclusion

Despite clinical practice guidelines recommending antiplatelet therapy for patients with diabetes, there are still many eligible patients not receiving this beneficial therapy, particularly patients under 65, women, and patients without known CVD. Effective methods to increase antiplatelet use should be considered at the national, community, practice and provider level.

## References

[B1] Moss SE, Klein R, Klein BE (1991). Cause-specific mortality in a population-based study of diabetes. Am J Public Health.

[B2] Turner R, Cull C, Holman R (1996). United Kingdom Prospective Diabetes Study 17: a 9-year update of a randomized, controlled trial on the effect of improved metabolic control on complications in non-insulin-dependent diabetes mellitus. Ann Intern Med.

[B3] Haffner SM, Lehto S, Ronnemaa T, Pyorala K, Laakso M (1998). Mortality from coronary heart disease in subjects with type 2 diabetes and in nondiabetic subjects with and without prior myocardial infarction. N Engl J Med.

[B4] Kannel WB, McGee DL (1979). Diabetes and cardiovascular disease. The Framingham study. Jama.

[B5] ETDRS I (1992). Aspirin effects on mortality and morbidity in patients with diabetes mellitus. Early Treatment Diabetic Retinopathy Study report 14. ETDRS Investigators. Jama.

[B6] Gaspoz JM, Coxson PG, Goldman PA, Williams LW, Kuntz KM, Hunink MG, Goldman L (2002). Cost effectiveness of aspirin, clopidogrel, or both for secondary prevention of coronary heart disease. N Engl J Med.

[B7] Harpaz D, Gottlieb S, Graff E, Boyko V, Kishon Y, Behar S (1998). Effects of aspirin treatment on survival in non-insulin-dependent diabetic patients with coronary artery disease. Israeli Bezafibrate Infarction Prevention Study Group. Am J Med.

[B8] Nobles-James C, James EA, Sowers JR (2004). Prevention of cardiovascular complications of diabetes mellitus by aspirin. Cardiovasc Drug Rev.

[B9] (2002). Aspirin for the primary prevention of cardiovascular events: recommendation and rationale. Ann Intern Med.

[B10] Hennekens CH, Dyken ML, Fuster V (1997). Aspirin as a therapeutic agent in cardiovascular disease: a statement for healthcare professionals from the American Heart Association. Circulation.

[B11] ADA (1997). Aspirin therapy in diabetes. American Diabetes Association. Diabetes Care.

[B12] Rolka DB, Fagot-Campagna A, Narayan KM (2001). Aspirin use among adults with diabetes: estimates from the Third National Health and Nutrition Examination Survey. Diabetes Care.

[B13] Persell SD, Baker DW (2004). Aspirin use among adults with diabetes: recent trends and emerging sex disparities. Arch Intern Med.

[B14] Harrington RA, Becker RC, Ezekowitz M, Meade TW, O'Connor CM, Vorchheimer DA, Guyatt GH (2004). Antithrombotic therapy for coronary artery disease: the Seventh ACCP Conference on Antithrombotic and Thrombolytic Therapy. Chest.

[B15] McFarlane SI, Jacober SJ, Winer N, Kaur J, Castro JP, Wui MA, Gliwa A, Von Gizycki H, Sowers JR (2002). Control of cardiovascular risk factors in patients with diabetes and hypertension at urban academic medical centers. Diabetes Care.

[B16] Stafford RS (2000). Aspirin use is low among United States outpatients with coronary artery disease. Circulation.

[B17] MacLean CD, Littenberg B, Gagnon MS, Reardon M, Turner PD, Jordan C (2004). The Vermont Diabetes Information System (VDIS): Study Design and Subject Recruitment for a Cluster Randomized Trial of a Decision Support System in a Regional Sample of Primary Care Practices. Clinical Trials.

[B18] ADA (2004). Aspirin Therapy in Diabetes. Diabetes Care.

[B19] CAPRIE (1996). A randomised, blinded, trial of clopidogrel versus aspirin in patients at risk of ischaemic events (CAPRIE). CAPRIE Steering Committee. Lancet.

[B20] Tran H, Anand SS (2004). Oral antiplatelet therapy in cerebrovascular disease, coronary artery disease, and peripheral arterial disease. Jama.

[B21] Mehta SR, Yusuf S (2003). Short- and long-term oral antiplatelet therapy in acute coronary syndromes and percutaneous coronary intervention. J Am Coll Cardiol.

[B22] Antithrombotic_Trialists'_Collaboration (2002). Collaborative meta-analysis of randomised trials of antiplatelet therapy for prevention of death, myocardial infarction, and stroke in high risk patients. BMJ.

[B23] Engelgau MM, Geiss LS, Saaddine JB, Boyle JP, Benjamin SM, Gregg EW, Tierney EF, Rios-Burrows N, Mokdad AH, Ford ES, Imperatore G, Narayan KM (2004). The evolving diabetes burden in the United States. Ann Intern Med.

[B24] Sacco M, Pellegrini F, Roncaglioni MC, Avanzini F, Tognoni G, Nicolucci A (2003). Primary prevention of cardiovascular events with low-dose aspirin and vitamin E in type 2 diabetic patients: results of the Primary Prevention Project (PPP) trial. Diabetes Care.

[B25] Short D, Frischer M, Bashford J, Ashcroft D (2003). Why are eligible patients not prescribed aspirin in primary care? A qualitative study indicating measures for improvement. BMC Fam Pract.

[B26] Tecce MA, Dasgupta I, Doherty JU (2003). Heart disease in older women. Gender differences affect diagnosis and treatment. Geriatrics.

[B27] Ridker PM, Cook NR, Lee IM, Gordon D, Gaziano JM, Manson JE, Hennekens CH, Buring JE (2005). A randomized trial of low-dose aspirin in the primary prevention of cardiovascular disease in women. N Engl J Med.

[B28] Diener HC, Ringleb P (2002). Antithrombotic Secondary Prevention After Stroke. Curr Treat Options Cardiovasc Med.

[B29] Faulkner LA, Schauffler HH (1997). The effect of health insurance coverage on the appropriate use of recommended clinical preventive services. Am J Prev Med.

[B30] Saaddine JB, Engelgau MM, Beckles GL, Gregg EW, Thompson TJ, Narayan KM (2002). A diabetes report card for the United States: quality of care in the 1990s. Ann Intern Med.

[B31] (2000). Improvement of Cardiac Outcomes in Kaiser Permanente of Ohio. The Permanente Journal.

[B32] Gaede P, Vedel P, Larsen N, Jensen GV, Parving HH, Pedersen O (2003). Multifactorial intervention and cardiovascular disease in patients with type 2 diabetes. N Engl J Med.

[B33] Faragon JJ, Waite NM, Hobson EH, Seoldo N, VanAmburgh JA, Migden H (2003). Improving aspirin prophylaxis in a primary care diabetic population. Pharmacotherapy.

[B34] Haggerty SA, Cerulli J, Zeolla MM, Cottrell JS, Weck MB, Faragon JJ (2005). Community pharmacy Target Intervention Program to improve aspirin use in persons with diabetes. J Am Pharm Assoc (Wash DC).

[B35] Filippi A, Sabatini A, Badioli L, Samani F, Mazzaglia G, Catapano A, Cricelli C (2003). Effects of an automated electronic reminder in changing the antiplatelet drug-prescribing behavior among Italian general practitioners in diabetic patients: an intervention trial. Diabetes Care.

[B36] Krein SL, Vijan S, Pogach LM, Hogan MM, Kerr EA (2002). Aspirin use and counseling about aspirin among patients with diabetes. Diabetes Care.

